# Posterior Circulation Acute Stroke Prognosis Early Computed Tomography Score Using Hypointense Vessels on Susceptibility Weighted Imaging Independently Predicts Outcome in Patients with Basilar Artery Occlusion

**DOI:** 10.1371/journal.pone.0132587

**Published:** 2015-07-15

**Authors:** S. Mundiyanapurath, M. Möhlenbruch, P. A. Ringleb, J. Bösel, W. Wick, M. Bendszus, A. Radbruch

**Affiliations:** 1 Department of Neurology, University Hospital Heidelberg, Heidelberg, Germany; 2 Department of Neuroradiology, University Hospital Heidelberg, Heidelberg, Germany; 3 German Cancer Research Center, Department of Radiology, Heidelberg, Germany; Julius-Maximilians-Universität Würzburg, GERMANY

## Abstract

**Purpose:**

Appearance of hypointense vessels on susceptibility weighted imaging (SWI) has been reported to correlate with outcome in patients with ischemia of the anterior circulation. This study investigates the correlation between the appearance of hypointense vessels on SWI after recanalization therapy and outcome in patients with basilar artery occlusion.

**Methods:**

Patients with basilar artery occlusion who were treated with endovascular recanalization or intravenous alteplase and received an MRI including SWI after therapy were retrieved from the hospital database for retrospective analysis. Posterior circulation Acute Stroke Prognosis Early Computed Tomography Score (pcASPECTS) was calculated based on regions displaying hypointense vessels on SWI and compared to lesions on diffusion weighted imaging (DWI). Subsequently, SWI based pcASPECTS was correlated with outcome determined with modified Rankin Scale (mRS), categorized as favorable outcome (mRS 0-2) or unfavorable outcome (3-6).

**Results:**

Twenty-two MRI of patients with basilar artery occlusion were analyzed. In seven out of eight areas of the pcASPECTS hypointense vessels on SWI were significantly correlated to areas of restricted diffusion on DWI. In univariate analysis median pcASPECTS on SWI was significantly higher in patients with favorable outcome (7.5 vs. 5, p=0.02). In a multivariate analysis pcASPECTS on SWI was an independent predictor of favorable outcome (OR 2.02; CI [1.02;3,99]; p=0.04).

**Conclusion:**

pcASPECTS based on hypointense vessels on SWI after therapy predicts outcome in patients with basilar artery occlusion and might potentially be used as an additional imaging biomarker in the management of patients with stroke in the posterior circulation. This needs to be confirmed in larger prospective clinical trials.

## Introduction

Stroke following basilar artery occlusion is a rare disease with high fatality and morbidity [1]. Therapeutic approaches include endovascular recanalization and intravenous or intraarterial alteplase. The selection of patients that benefit from these therapies is challenging and studies that aimed at identifying these patients focused mainly on perfusion weighted imaging in several small case series [2–4]. Currently, studies with larger patient numbers do not exist due to the low incidence of basilar artery occlusion. Unlike the findings in the anterior circulation, where the mismatch between perfusion MRI and diffusion MRI is supposed to identify the tissue at risk, this concept is not applicable to the posterior circulation. This is partly caused by technical limitations of the perfusion MRI, particularly the low spatial resolution of perfusion weighted magnetic resonance imaging (PW-MRI). Although a clear mismatch often cannot be defined, trials have shown that it is possible to make a prognosis based on diffusion weighted imaging (DWI) and computed tomography angiography source images using the posterior circulation Acute Stroke Prognosis Early Computed Tomography Score (pcASPECTS) [5, 6]. Other parameters such as early recanalization, site of occlusion (proximal, mid-basilar or distal basilar artery) and successful recanalization have also been shown to be predictors of outcome [7]. However, these prognostic factors have not been validated in large case series or randomized trials and are currently not used in the selection of patients eligible for therapy [7]. Due to the frequent fatal course, withdrawal of life support is discussed frequently in patients with basilar artery occlusion. Thus, clinicians need as much information as possible on how to predict outcome in this disease.

Recently, susceptibility weighted imaging (SWI) has come into focus for scientists studying cerebral ischemia. SWI is a new MRI technique that leads to an increased contrast and conspicuity of T2*-lesions, by combining the phase mask with the corresponding gradient echo magnitude map [8–10]. The additional phase information provides an increased sensitivity for deoxyhemoglobin. SWI has been used to detect intracranial hemorrhage or microbleeds for many years [10]. More recently, signal changes of vessels on SWI were reported to occur in ischemic stroke [11–13]. It is supposed that the prominent cortical vessels on SWI are signs of collateral flow in the tissue at risk, caused by an increased concentration of deoxyhemoglobin[14]. For ischemic stroke in the anterior circulation it was recently reported that a mismatch calculated on the basis of diffusion weighted imaging and areas displaying hypointense vessels on SWI may predict outcome [11, 13]. Furthermore, it was reported that the appearance of deep medullary veins in the anterior circulation is associated with poor outcome [15].

In the current study, we investigated whether hypointense vessels on SWI in patients with basilar artery occlusion are associated with restricted diffusion on diffusion weighted images (DWI) and outcome.

## Methods

### Patient selection

The study was approved by the ethics committee of the University of Heidelberg, Germany (statement S-330/2012). Due to the retrospective nature of this study the ethics committee did not require subsequent informed written consent of the included patients. Analysis was performed after pseudonymization.

All patients with acute basilar artery occlusion that were treated with endovascular recanalization or intravenous alteplase and received an MRI including SWI and their baseline characteristics as well as information on treatment and outcome (age, gender, National Institute of Health Stroke Scale Score = NIHSSS on admission, modified Rankin Score (mRS) after three months, symptom-onset-to-recanalization time, symptom-to-thrombolysis-time, stent device used, usage of glycoprotein IIbIIIa (GpIIbIIIa) antagonists, suspected etiology of stroke, site of occlusion and result of recanalization) were collected from the hospital database. The Thrombolysis in Cerebral Infarction (TICI) Score was applied to grade reperfusion. Different parameters of seven of the included patients were reported before [5].

Clinical outcome was assessed three months after stroke by an investigator not blinded to the treatment but to this analysis by a standardized telephone-interview or an inpatient-visit using the modified Rankin Scale (mRS). Good outcome was defined as a mRS score of 0 to 2 on this scale, reflecting to be able to live independently. This definition is commonly used in stroke trials [16].

### Image Acquisition

Images were acquired during routine clinical diagnostics using a 3 Tesla MR system (Magnetom Tim Trio or Verio with identical technical parameters, Siemens Healthcare, Erlangen, Germany) with a 12-channel head-matrix coil. Whole brain SWI data were recorded with a 3D, fully flow-compensated gradient echo sequence using the following parameters: echo time (TE) 19.7 ms, repetition time (TR) 27 ms, flip angle (FA) 15°, FoV 230 x 230 mm, slice thickness 3 mm, pixel spacing 0.72 and an acquisition time of 2:16 min. The GRE magnitude and phase images were converted into SW images by the MR-scanner software. DWI was performed using a single-shot spin-echo (SE) echo-planar sequence with the following parameters: TE = 90 ms, TR = 5300 ms, FA = 90°, slice thickness = 5 mm. Diffusion sensitizing gradients were applied sequentially in the x, y, and z directions with b-values of 0 and 1200 s/mm2. Apparent diffusion coefficient (ADC) trace maps were created automatically using the above mentioned software, supplied by the manufacturer.

### Image Analysis

Images were analyzed using Centricity PACS Radiology Workstation (GE Healthcare, Barrington, Illinois, USA) and were assessed on monitors for diagnostic purposes. Pathological hypointense vessels were scored in the brainstem (midbrain and pons separately), cerebellum, thalamus and the posterior cerebral artery (PCA) territory on SWI by visual inspection. DWI signal was scored in the same territories. The territory was scored as affected by ischemia when at least one clearly hypointense vessel could be identified. The scoring was done separately by two readers (S.M. and A.R.) who were blinded to the outcome parameters. Any case of disagreement was resolved by consensus reading. SWI was scored prior to DWI to avoid a reading bias. pcASPECTS was calculated as described before [6].

### Statistical Analysis

Statistical analysis was conducted using Microsoft Excel Version 2003 and IBM SPSS Version 21. Correlations were calculated using the Pearsons bivariate correlation coefficient. Univariate analysis was performed using Mann-Whitney-U and Chi-square / Fisher exact test depending on the distribution and the size of the tested groups. Pretesting for normal distribution was not performed to avoid error accumulation [17]. An α-Level of 0.05 was chosen. Multivariate analysis was carried out applying likelihood-ratio test based stepwise forward selection within binary logistic regression models where variables were included if the related p-value was above 0.05. Two-sided p-values are reported throughout.

## Results

Twenty-two MRI of patients with basilar artery occlusion in the time period from 2009–2014 were retrieved. One patient had to be excluded due to severe motion artifacts. In all patients the MRI was performed after the intervention (mean time till MRI 4 h 54 min, standard deviation 1h 53 min). Four patients were treated with intravenous alteplase, four patients with mechanical recanalization and 14 patients with intravenous alteplase and mechanical recanalization.

**Fig 1:**
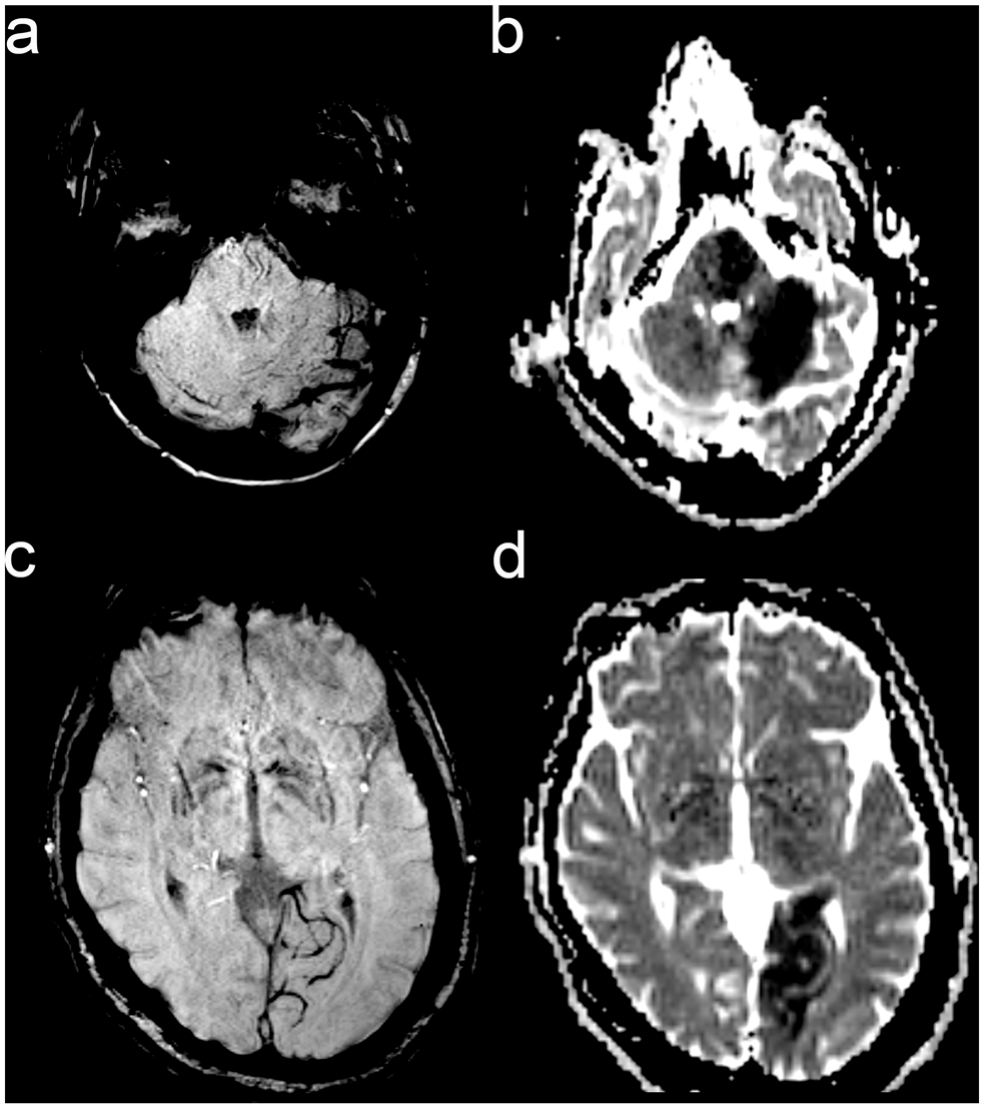
SWI changes in basilar artery occlusion. MRI was performed in two different patients after endovascular recanalization in basilar artery occlusion. Hypointense and enhanced vessels are displayed in the brain stem and cerebellum of one patient (a) and in the PCA-territory of another patient (c). Corresponding DWI changes appear on apparent diffusion coefficient maps in the same region (b, d).

Comparing the group of patients with favorable outcome (mRS 0–2) to the one with unfavorable outcome (mRS 3–6), there was no significant difference in terms of age, gender, etiology of the occlusion, site of occlusion, usage of GpIIbIIIa-antagonist, mode of treatment (intravenous alteplase, endovascular recanalization or their combination) and stent implantation (Table 1). Hypointense, prominent vessels displayed in all regions of the posterior circulation territory (Fig 1). Appearance of vessels in the territory of the PCA were similar to the previously described hypointense cortical vessels of the anterior circulation[13]. Particularly, the vessels in the cerebellum and in the brainstem resembled the deep medullary veins that were described previously in the anterior circulation [18]. However, vessel pathologies tended to be more subtle compared to the anterior circulation, impeding the determination of a clear territory of hypointense cortical vessels and the concrete calculation of a SWI/DWI mismatch volume (Fig 1).

**Table 1:** Baseline and clinical characteristics as well as significant differences in univariate analysis in patients with favorable (mRS 0–2) and unfavorable outcome (mRS 3–6).

	Favorable Outcome	Unfavorable Outcome	p-value
N	6	16	
Mean Age (SD;range)	61.5 (13.0;47–79)	69.5 (9.2; 51–85)	0.23
Female Gender	2 (33%)	5 (31.3%)	0.65
Etiology			0.73
- Cardioembolic	3 (50%)	6 (37.5%)	
- Atherosclerosis	1 (16.7%)	6 (37.5%)	
- Dissection	1 (16.7%)	1 (6.3%)	
- Unclear	1 (16.7%)	3 (18.8%)	
Glycoprotein IIbIIIa-Antagonist	2 (33%)	7 (43.8%)	0.48
Site of occlusion			0.51
- Proximal	1 (17%)	4 (25%)	
- Mid-basilar	3 (50%)	7 (44%)	
- Distal	2 (33%)	5 (31%)	
Permanent stent	3 (50%)	13 (81.3%)	0.16
Complete recanalization (TICI 2b or 3)	3 (50%)	8 (50%)	1.00
**Median pcASPECTS (range)**	**7.5 (4–10)**	**5 (1–8)**	**0.02**
**Median NIHSSS on admission (range)**	**17 (6–36)**	**38 (7–39)**	**0.02**
**Mean symptom-onset-to-recanalization time in minutes (range)**	**133 (80–231)**	**307 (110–750)**	**0.05**

There was a significant correlation between SWI and DWI lesions for all but the left PCA territory (Pearson correlation: left thalamus 0.585, p = 0.003; right thalamus 0.776 p<0.001, brainstem 0.631, p = 0.001; left cerebellum 0.826, p<0.001; right cerebellum 0.523, p = 0.01; left PCA territory 0.28, p = 0.196; right PCA territory 0.562, p = 0.05). pcASPECTS with DWI was lower than pcASPECTS with SWI (4.5 [IQR 2.0;7.0] vs. 6 [IQR 3.8;7.3], p = 0.048). In an univariate analysis the following parameters were significantly different in patients with favorable compared to patients with unfavorable outcome: median pcASPECTS (7.5 vs. 5, p = 0.02), median NIHSSS on admission (17 vs. 38, p = 0.02) and mean symptom-onset-to-recanalization time (133 min vs. 307 min, p = 0.05) (Table 1). In the multivariate binary logistic stepwise regression including age, degree of recanalization, pcASPECTS, NIHSSS and symptom-onset-to-recanalization time, only pcASPECTS was an independent predictor of outcome (Odds Ratio 2.02, p = 0.043, confidence interval [1.02;3.99]).

To compare the diagnostic value of the pcASPECTS based on DWI or SWI, we performed a ROC-curve analysis. Area under the curve was 0.833 for pcASPECTS using SWI, 0.859 using DWI and 0.865 using the mean of both scores.

## Discussion

The current study provides evidence that pcASPECTS based on hypointense vessels on SWI after recanalization therapy is an independent predictor of outcome in patients with basilar artery occlusion. Furthermore, we found a significant correlation of areas with hypointense vessels on SWI and areas with restricted diffusion on DWI in 7 out of 8 assessed areas in pcASPECTS.

After basilar artery occlusion, patients often suffer from severe neurological deficits and are treated on neurological intensive care units or stroke units. In this situation, MRI is often used to estimate a patient’s prognosis. Since a fatal prognosis might result in the decision for a palliative care approach, reliable imaging parameters for outcome prediction are obviously needed.

Within daily clinical decision making, DWI is the predominantly used MRI sequence for outcome prediction in patients with basilar artery occlusion and its predictive value has been demonstrated in a previously published study [5]. However, it has also been reported that restrictions on DWI maps are potentially reversible causing a certain degree of uncertainty in the estimated prognosis [19–21].

SWI might therefore be used as an additional imaging biomarker to verify the proposed diagnosis based on DWI. In our cohort pcASPECTS based on SWI did not show a relevant additional diagnostic accuracy compared to pcASPECTS used with DWI in the ROC-curve analysis. Nevertheless, in case of DWI lesion reversal, SWI might give additional information about the expected prognosis. Our findings showed a correlation between areas with hypointense vessels on SWI and areas with restricted diffusion on DWI assessed for pcASPECTS score besides the left PCA territory. It is highly likely that the missing correlation for hypointense vessels on SWI and diffusion restrictions on DWI in the left PCA territory can be explained by a bias caused by the low number of included patients. As expected, NIHSSS on admission and symptom-onset-to-recanalization time were different in patients with favorable and unfavorable outcome as well. However, multivariate analysis showed that pcASPECTS based on SWI could independently predict outcome in patients with basilar artery occlusion. The proportion of patients with complete recanalization was not significantly different between patients with favorable and unfavorable outcome, which can be explained by an imbalance in symptom-onset-to-recanalization time. Patients that had a TICI score of 2b or 3 in the group of patients with unfavorable outcome were recanalized after a median symptom-onset-to-recanalization time of 343 (IQR 200;495) minutes.

The pathophysiological explanation for the hypointense vessels on SWI in the posterior circulation equals the established concept of prominent cortical veins on SWI in the anterior circulation. It is assumed that the hypointense signal within cortical veins on SWI in the anterior circulation is caused by an uncoupling between oxygen supply and demand in the hypoperfused tissue that results in a relative increase of deoxyhemoglobin and a relative decrease of oxyhemoglobin [22]. In case of artery occlusion the reduced blood flow within the affected territory leads to an increased oxygen extraction fraction that causes an increase of the concentration of deoxyhemoglobin within the venous system. This increase in deoxyhemoglobin causes susceptibility changes and finally the prominent hypointense signal within veins on SWI [23].

However, differences to the findings of SWI changes in the anterior circulation have to be acknowledged. Generally, the visibility of hypoperfused areas on SWI depends on the existence and homogenous distribution of vessels that present with a diameter above the limit of detection on SWI. In the anterior circulation, cortical veins or deep medullary veins present such vessels. Therefore hypoperfused areas can be easily identified by the presence of hypointense veins and used as basis for a mismatch calculation.

In contrast, identification of hypoperfused areas on SWI in the posterior circulation is in our experience more challenging due to the following two reasons: especially in the cerebellum the distribution of cortical veins is more heterogeneous than in the cerebral cortex and prominent vessels with a large diameter also appear in regions that are not affected by ischemia; furthermore, cortical vessels surrounding the brainstem or the thalamus are often hard to identify due to the small vessel caliber of vessels in these regions [24].

Even though this lack of homogenous distribution of cortical vessels with a diameter above detection in the posterior circulation often impedes the identification and quantification of a tissue at risk, our study proved that hypointense vessels within the tissue can be used as an imaging biomarker in the posterior circulation when they are scored according to the pcASPECTS.

Limitations of this study have to be acknowledged and are caused by the small patient number and the retrospective design, which makes our study prone to selection bias. We analyzed the excluded patients regarding baseline and clinical parameters and compared them to the study cohort (S1 Table). Only the percentage of patients treated with glycoprotein IIbIIIa-antagonist and permanent stent was significantly different. The small patient number especially affects the multivariate analysis, which has limited power with four variables included. Regarding the result of recanalization, data was not available for the 4 patients that were treated with intravenous alteplase alone. Furthermore, our study does not assess the timely development of SWI alterations. Therefore we cannot exclude that SWI alterations disappear in patients presenting reversible restrictions on DWI. In addition, SWI changes before therapy could not be assessed, which might even be more important for the assessment of the potential benefit from recanalization therapy. Further research in a prospective approach is certainly needed to clarify this question.

In conclusion, we showed that the assessment of SWI using pcASPECTS in patients with basilar artery occlusion correlates with outcome as well as with DWI lesions. The predictive value of the pcASCPECTS based on SWI–if replicated in larger patient cohorts in future studies–might potentially help to assess prognosis and guide therapeutic decisions in patients with basilar artery occlusion.

## Supporting Information

S1 Table Comparison of baseline and clinical characteristics between patients included in the study and patients who were excluded. Data are presented as median with IQR or percentage. Mann-Whitney-U test, Chi-square test or Fisher exact test were applied depending on the distribution and the size of the tested groups.
(DOCX)
