# Supplementary material for: Posterior Circulation Acute Stroke Prognosis Early Computed Tomography Score Using Hypointense Vessels on Susceptibility Weighted Imaging Independently Predicts Outcome in Patients with Basilar Artery Occlusion
Source: PLoS One. 2015 Jul 15;10(7):e0132587. doi: 10.1371/journal.pone.0132587 (PMC4503629; doi:10.1371/journal.pone.0132587)
Supplement: S1 Table — Data are presented as median with IQR or percentage. Mann-Whitney-U test, Chi-square test or Fisher exact test were applied depending on the distribution and the size of the tested groups. (DOCX) [file pone.0132587.s001.docx]

**S1 Table: Comparison of baseline and clinical characteristics between patients included in the study and patients who were excluded.** Data are presented as median with IQR or percentage. Mann-Whitney-U test, Chi-square test or Fisher exact test were applied depending on the distribution and the size of the tested groups

|  | Study population (n=22) | Excluded  (n=55) | p-value |
| --- | --- | --- | --- |
| Mean age (SD;range) | 67.3 (10.7;47-85) | 71.8 (14.5;20-95) | 0.095 |
| Female gender | 32 % | 46% |  |
| Mean symptom onset-to-recanalization time (SD; range) | 259.3 (186.3; 80-750) | 175.5 (144.6; 70-590) | 0.097 |
| Median NIHSSS on admission (range) | 35 (6-39) | 27 (4-39) | 0.188 |
| Treatment modality   - Intravenous Alteplase - Intraarterial therapy - Intravenous and intraarterial therapy | 18%  18%  64% | 11%  26%  64% | 0.610 |
| Glycoprotein IIbIIIa-antagonist | 41% | 16% | 0.015 |
| Permanent stent | 72% | 20% | <0.001 |
| Complete recanalization | 50% | 47% | 0.249 |
| Favorable outcome | 27% | 33% | 0.606 |
| Stroke etiology   - Cardioembolic - Atherosclerosis - Dissection - Unclear | 41%  32%  9%  18% | 55%  31%  4%  11% | 0.483 |
